# When Obligate Partners Melt Down

**DOI:** 10.1128/mBio.01904-16

**Published:** 2016-11-15

**Authors:** Nancy A. Moran

**Affiliations:** Department of Integrative Biology, University of Texas at Austin, Austin, Texas, USA

## Abstract

Insect hosts derive benefits from their obligate symbionts, including nutrient supplementation and the ability to colonize otherwise inhospitable niches. But long-term symbionts sometimes also limit the ecological range of their hosts; in particular, they are often more temperature sensitive than the hosts themselves. Even small increases in average temperature, comparable to those occurring under current conditions of climate change, can kill symbionts and, with them, their hosts. In some cases, limitations imposed by obligate symbionts may help to counter the spread of invasive pests, but they also contribute to contractions in populations and geographic ranges of invertebrate species.

## COMMENTARY

The consequences of continuing climate change are complex and are sure to bring surprises. Most people are more concerned about the effects on property values in Miami than about potential declines in insect populations. But insects, and other invertebrates, are crucial links in all ecosystems, and changes in their populations and geographic distributions will have unpredictable consequences. In a recent article, Kikuchi et al. (1) demonstrate that rather small increases in average temperature resulted in catastrophic developmental changes in a crop pest, the southern green stinkbug (*Nezara viridula*). The new finding is consistent with previous field documentation of northward shifts of this insect species within Japan (2). Remarkably, Kikuchi et al. show that these defects are due not to direct effects on insect development but to detrimental effects on the obligate gammaproteobacterial symbionts that reside in the midgut. These symbionts have previously been shown to be required for normal host development. Mother stinkbugs ensure colonization of their progeny by smearing eggs with feces containing bacterial cells that are subsequently ingested by the hatchlings. That new study showed that elevated temperatures have the same effects as administration of antibiotics in severely depressing symbiont titers and that heat treatment and antibiotic treatment have the same harmful effects on insect development.

No one is likely to mourn the decline of this particular stinkbug, which is an invasive pest of soybean and other crops. But the southern green stinkbug is but one example of many thousands of insects and other invertebrates that rely on microbial associates for normal function and development. The roots of these symbiotic associations often can be traced to the very deep evolutionary past. For example, the symbiosis between *Buchnera aphidicola* and its sap-feeding aphid hosts dates to over 100 million years ago, and symbioses of leafhoppers and cicadas are even older (3). The evolutionary basis for most such cases of obligate symbiosis stem from mutual advantages involving nutrient provisioning by the symbiont to the host; for example, *Buchnera* makes essential amino acids that are rare in the phloem sap diet of aphids. But long coevolution has resulted in many additional dependencies of hosts on symbionts, and symbionts are often required for normal host development even when the limiting nutrients are supplemented experimentally.

Why should symbiont cells succumb to heat stress more readily than the cells and tissues of their hosts? Most insect symbionts resemble mitochondria in being strictly maternally inherited. In the long run, the resulting clonal population structure causes degenerative evolution of genes and genomes. This is because small population sizes and clonality make deleterious mutations more likely to persist and become fixed, a phenomenon referred to as Muller’s Ratchet, after H. J. Muller, who studied many aspects of mutation in populations (4). In contrast, typical bacterial populations have the advantages of being very large and undergoing some genetic recombination, enabling them to efficiently eliminate deleterious mutations. One consequence of the population structure of maternally transmitted symbionts is the elimination of any genes that are not absolutely essential. In fact, all of the tiniest known bacterial genomes correspond to obligate symbionts of invertebrates (5). But degenerative evolution also affects the essential protein-coding genes that are retained in symbiont genomes. Most new mutations in coding genes result in a change in an encoded amino acid, and most random changes in an amino acid in a functional protein decrease the thermal stability of that protein. In obligate symbiont populations, selection is less able to eliminate such mutations, yielding not only tiny genomes but also proteins that easily melt. The resulting protein misfolding and aggregation are the key cellular effects of heat stress.

A repeated observation from proteomic or transcriptomic studies of obligate symbionts is that these organisms express heat shock proteins such as chaperonin (GroEL) at exceptionally high levels under all conditions (6). This investment in machinery for protein refolding appears to be a compensation for the buildup of deleterious mutations that compromise protein thermal stability (7).

A study on *Buchnera* shed some light on the role of protein stability in symbiont susceptibility to heat stress. A single point mutation in the *Buchnera* promoter for *ibpA*, which encodes a universally distributed small heat shock protein, virtually eliminated the transcriptional response to heat, and this had drastic negative consequences both for *Buchnera* titers and for the growth and fecundity of aphid hosts following thermal challenge (8). When the heat-sensitive *Buchnera* lineage was eliminated from an aphid matriline and replaced by a heat-tolerant *Buchnera* genotype, aphid fitness following heat exposure rebounded (9).

One interesting issue is whether dependence on degenerative obligate symbionts has been a factor in the extinction of host lineages as the Earth has undergone shifts in climate over evolutionary time. The first step toward extinction is a limited geographic range. It is very likely that the geographic ranges of hosts are sometimes curtailed by the heat intolerance of their obligate symbionts, with the study by Kikuchi et al. (1) providing one example. And an extension of this issue is whether dependence on obligate symbionts will speed range shifts, declines, and extinctions going forward as temperatures rise in many regions.

Insects and other invertebrates play central roles in food webs. Examples of insects dependent on maternally transmitted symbionts can be found in every ecological guild. The detritivores, which govern turnover in plant biomass and thus impact terrestrial ecosystems and biogeochemical cycles, include termites, cockroaches, and earthworms, all of which depend on obligate bacterial symbionts. Populations of symbiont-dependent herbivores, such as leafhoppers and aphids, are a primary food supply for diverse types of predators and parasites, from tiny wasps to birds and bats. Marine invertebrates, such as some clams, sponges, and flatworms, also have ancient maternally transmitted symbionts (10–12), and these symbioses will likely affect their ability to respond to changing ocean environments.

Although essentially all animals have some dependence on microorganisms that play a part in normal development, not all have obligate, maternally transmitted symbionts prone to genome degradation and temperature sensitivity. For example, the important invasive vector species *Aedes aegyptii* and *Aedes albopictus* do not have routine associations with vertically transmitted bacteria, although bacteria acquired from the environment play essential roles in mosquito development (13). In fact, *A. albopictus* is an example of a heat-loving insect that is expanding its range progressively northward with changes in climate, in the process expanding its potential as a vector of arboviruses such as dengue virus and Zika virus. Likewise, bobtail squid depend on *Vibrio fischeri*, a symbiont that does not have a degenerative genome (14), reflecting the fact that it is environmentally rather than maternally transmitted and thus has a normal bacterial population structure. Leaf cutter ants depend on fungal symbionts to digest harvested plant tissues, and these symbionts are sometimes transferred among colonies of the same and different ant species (15). In *Atta texana*, a leaf cutter ant at the northernmost edge of leaf cutter distributions, populations experiencing different climatic conditions adopt fungal symbionts with corresponding temperature preferences, and this plasticity in symbiotic association enables *A. texana* to have a large geographic range (16). So, while we can reasonably expect symbioses to affect the responses of invertebrate populations to climate change, the effects vary according to the nature of the particular association.

Grain weevils (*Sitophilus*) are important pests of stored products and depend on obligate heat-sensitive symbionts (17). Recent discoveries suggest that quite a large proportion of weevils and other beetles may have vertically transmitted obligate symbionts (18). This expands the potential importance of symbiont heat sensitivity as a critical factor determining effects of climate change: beetles comprise an estimated 40% of animal diversity and occupy an immense range of ecological roles, from specialized herbivores of leaves, seeds, wood, and fruits to fungivores, detritivores, and predators. Potentially, temperature-sensitive symbionts will play a big role in future range shifts and extinctions.

Unfortunately, we have few data on how invertebrate populations have been affected by climate change, habitat loss, and other anthropogenic shifts in recent decades. For insects in particular, we have not yet come close to describing the existing diversity (19), and, to most people, the fate of obscure insect species is not a major concern. The main cases for which we have any information or concern involve large and attractive insects, such as certain bumblebees and Monarch butterflies, whose populations appear to be rapidly declining (20, 21), or threatening insects, such as *A. albopictus*, which has expanded its geographic range in the last few decades (22). But the number of insect species is enormous, and only a few are pests, so shifts in population sizes or distributions go unremarked.

Though they do not receive as much attention as birds and mammals, insects make up a bigger part of native biodiversity and of the coevolved biological systems that are disappearing. Most herbivorous insects feed on a very limited set of native plant species (23) and are thus highly dependent on preservation or regeneration of the habitat where those plants live. Specialized interactions between pairs of species, such as particular pollinators and the plants they service, have been noted as being particularly vulnerable to climate change (24). Dependence on temperature-sensitive symbionts adds to the other factors that threaten many invertebrate populations. For example, some populations of periodical cicadas of North America (*Magicicada*) are already decreasing or even disappearing and have ecological requirements and distributions that make range shifts unlikely (25). Added to this vulnerability is the dependence of cicadas on a pair of bacterial symbionts, “*Candidatus* Sulcia mulleri” (*Bacteroidetes*) and “*Candidatus* Hodgkinia cicadicola” (*Alphaproteobacteria*), both of which have highly degenerative genomes (26). Dependence on these symbionts may be the “Achilles heel” of cicadas, rendering them even less able to sustain themselves in the face of changing thermal environments.

We lack systematic data on whether insect species with obligate symbionts are generally more susceptible to temperature shifts, but numerous case studies have suggested this possibility (27). Examples such as that provided by Kikuchi et al. show that symbiosis, in at least some cases, does play a key part in making hosts intolerant to environmental change.

## References

[1] Kikuchi Y, Tada A, Musolin DL, Hari N, Hosokawa T, Fujisaki K, Fukatsu T 2016 Collapse of insect gut symbiosis under simulated climate change. mBio 7:e01578-16. doi:10.1128/mBio.01578-16. 27703075 PMC5050343

[2] Musolin DL, Tougou D, Fujisaki K 2009 Too hot to handle? Phenological and life-history responses to simulated climate change of the southern green stink bug *Nezara viridula* (Heteroptera: Pentatomidae). Glob Change Biol 16:73–87. doi:10.1111/j.1365-2486.2009.01914.x.

[3] Bennett GM, Moran NA 2015 Heritable symbiosis: the advantages and perils of an evolutionary rabbit hole. Proc Natl Acad Sci U S A 112:10169–10176. doi:10.1073/pnas.1421388112. 25713367 PMC4547261

[4] Muller HJ 1964 The relation of recombination to mutational advance. Mutat Res 106:2–9. doi:10.1016/0027-5107(64)90047-8. 14195748

[5] Moran NA, Bennett GM 2014 The tiniest tiny genomes. Annu Rev Microbiol 68:195–215. doi:10.1146/annurev-micro-091213-112901. 24995872

[6] Baumann P, Baumann L, Clark MA 1996 Levels of Buchnera aphidicola chaperonin GroEL during growth of the aphid *Schizaphis graminum*. Curr Microbiol 32:279–285. doi:10.1007/s002849900050.

[7] Wernegreen JJ 2012 Mutualism meltdown in insects: bacteria constrain thermal adaptation. Curr Opin Microbiol 15:255–262. doi:10.1016/j.mib.2012.02.001. 22381679 PMC3590105

[8] Dunbar HE, Wilson AC, Ferguson NR, Moran NA 2007 Aphid thermal tolerance is governed by a point mutation in bacterial symbionts. PLoS Biol 5:e96. doi:10.1371/journal.pbio.0050096. 17425405 PMC1847839

[9] Moran NA, Yun Y 2015 Experimental replacement of an obligate insect symbiont. Proc Natl Acad Sci U S A 112:2093–2096. doi:10.1073/pnas.1420037112. 25561531 PMC4343100

[10] Kuwahara H, Yoshida T, Takaki Y, Shimamura S, Nishi S, Harada M, Matsuyama K, Takishita K, Kawato M, Uematsu K, Fujiwara Y, Sato T, Kato C, Kitagawa M, Kato I, Maruyama T 2007 Reduced genome of the thioautotrophic intracellular symbiont in a deep-sea clam, *Calyptogena okutanii*. Curr Biol 17:881–886. doi:10.1016/j.cub.2007.04.039. 17493812

[11] Webster NS, Taylor MW 2012 Marine sponges and their microbial symbionts: love and other relationships. Environ Microbiol 14:335–346. doi:10.1111/j.1462-2920.2011.02460.x. 21443739

[12] Gao ZM, Wang Y, Tian RM, Wong YH, Batang ZB, Al-Suwailem AM, Bajic VB, Qian PY 2014 Symbiotic adaptation drives genome streamlining of the cyanobacterial sponge symbiont “Candidatus Synechococcus spongiarum.” mBio 5:e00079-14. doi:10.1128/mBio.00079-14. 24692632 PMC3977351

[13] Coon KL, Vogel KJ, Brown MR, Strand MR 2014 Mosquitoes rely on their gut microbiota for development. Mol Ecol 23:2727–2739. doi:10.1111/mec.12771. 24766707 PMC4083365

[14] Ruby EG, Urbanowski M, Campbell J, Dunn A, Faini M, Gunsalus R, Lostroh P, Lupp C, McCann J, Millikan D, Schaefer A, Stabb E, Stevens A, Visick K, Whistler C, Greenberg EP 2005 Complete genome sequence of Vibrio fischeri: a symbiotic bacterium with pathogenic congeners. Proc Natl Acad Sci U S A 102:3004–3009. doi:10.1073/pnas.0409900102. 15703294 PMC549501

[15] Mikheyev AS, Mueller UG, Boomsma JJ 2007 Population genetic signatures of diffuse co-evolution between leaf-cutting ants and their cultivar fungi. Mol Ecol 16:209–216. doi:10.1111/j.1365-294X.2006.03134.x. 17181732

[16] Mueller UG, Mikheyev AS, Hong E, Sen R, Warren DL, Solomon SE, Ishak HD, Cooper M, Miller JL, Shaffer KA, Juenger TE 2011 Evolution of cold-tolerant fungal symbionts permits winter fungiculture by leafcutter ants at the northern frontier of a tropical ant-fungus symbiosis. Proc Natl Acad Sci U S A 108:4053–4056. doi:10.1073/pnas.1015806108. 21368106 PMC3053988

[17] Carvalho GA, Vieira JL, Haro MM, Corrêa AS, Ribon AO, de Oliveira LO, Guedes RN 2014 Pleiotropic impact of endosymbiont load and co-occurrence in the maize weevil *Sitophilus zeamais*. PLoS One 9:e111396. doi:10.1371/journal.pone.0111396. 25347417 PMC4210188

[18] Toju H, Tanabe AS, Notsu Y, Sota T, Fukatsu T 2013 Diversification of endosymbiosis: replacements, co-speciation and promiscuity of bacteriocyte symbionts in weevils. ISME J 7:1378–1390. doi:10.1038/ismej.2013.27. 23446834 PMC3695293

[19] Stork NE, McBroom J, Gely C, Hamilton AJ 2015 New approaches narrow global species estimates for beetles, insects, and terrestrial arthropods. Proc Natl Acad Sci U S A 112:7519–7523. doi:10.1073/pnas.1502408112. 26034274 PMC4475949

[20] Brower LP, Taylor OR, Williams EH, Slayback DA, Zubieta RR, Ramírez MI 2012 Decline of monarch butterflies overwintering in Mexico: is the migratory phenomenon at risk? Insect Conserv Divers 5:95–10021. doi:10.1111/j.1752-4598.2011.00142.x.

[21] Goulson D, Nicholls E, Botías C, Rotheray EL 2015 Bee declines driven by combined stress from parasites, pesticides, and lack of flowers. Science 347:1255957. doi:10.1126/science.1255957. 25721506

[22] Campbell LP, Luther C, Moo-Llanes D, Ramsey JM, Danis-Lozano R, Peterson AT 2015 Climate change influences on global distributions of dengue and Chikungunya virus vectors. Philos Trans R Soc Lond B Biol Sci 370:20140135. doi:10.1098/rstb.2014.0135. 25688023 PMC4342968

[23] Dyer LA, Singer MS, Lill JT, Stireman JO, Gentry GL, Marquis RJ, Ricklefs RE, Greeney HF, Wagner DL, Morais HC, Diniz IR, Kursar TA, Coley PD 2007 Host specificity of Lepidoptera in tropical and temperate forests. Nature 448:696–699. doi:10.1038/nature05884. 17687325

[24] Toby Kiers E, Palmer TM, Ives AR, Bruno JF, Bronstein JL 2010 Mutualisms in a changing world: an evolutionary perspective. Ecol Lett 13:1459–1474. doi:10.1111/j.1461-0248.2010.01538.x. 20955506

[25] Cooley JR, Marshall DC, Simon C 2013 At the limits: habitat suitability modelling of northern 17-year periodical cicada extinctions (Hemiptera: *Magicicada* spp.). Global Ecol Biogeogr 4:410–412.

[26] Moran NA, McCutcheon JP, Nakabachi A 2008 Genomics and evolution of heritable bacterial symbionts. Annu Rev Genet 42:165–190. doi:10.1146/annurev.genet.41.110306.130119. 18983256

[27] Corbin C, Heyworth ER, Ferrari J, Hurst GD 2016 Heritable symbionts in a world of varying temperature. Heredity (Edinb) doi:10.1038/hdy.2016.71. PMC5176117 27703153

